# Prevalence and factors affecting enuresis amongst primary school children

**DOI:** 10.4103/0970-1591.36703

**Published:** 2007

**Authors:** Avinash De Sousa, Hema Kapoor, Jyoti Jagtap, Mercilina Sen

**Affiliations:** Get Well Clinic and Nursing Home, Mumbai, India

**Keywords:** Enuresis, primary school children

## Abstract

**Aims and Objectives:**

The aim of the study was to establish the prevalence of enuresis in school children and to determine contributing factors along with treatment methods used in these children.

**Materials and Methods:**

The parents of 1473 children aged between 6-10 years completed a self-administered semi-structured questionnaire. Socio-demographic profiles, enuresis data, medical and psychiatric disorders and family stressors were collected. The data was analyzed and the results presented.

**Results:**

The response rate was 89.22%. The overall prevalence of enuresis was 7.61%. Enuresis was more common in boys. A positive family history of enuresis was seen in 28.57% children; 14.29% of the children had daytime wetting as well. Only 24.11% of the parents had taken their child to a doctor for the problem. Family stressors, significant birth history and lower socioeconomic status was present to a larger extent in the enuretic group. Scholastic backwardness was also an important factor in this group.

**Conclusions:**

This study reports on the prevalence of enuresis in school-going children and stresses on the need for parental education and awareness about this problem.

Enuresis is defined as an involuntary and undesirable bedwetting beyond the age of anticipated bladder control. It is seen worldwide across all races and cultures. It is a common problem amongst school children and the reported prevalence varies across studies. These inconsistencies often arise due to imprecise definitions.[1] Studies report the prevalence of enuresis as 12-25% amongst four-year-olds, 8-10% amongst eight-year-olds and 2-3% amongst 12-year-olds.[2]

Most school-based epidemiology studies on child and adolescent psychiatric disorders in India have focused on disorders of concern like learning disability, mental retardation and attention deficit hyperactivity disorder with little or no reference to enuresis. There have been only three published studies in India that have reported the prevalence of enuresis in school children with the range being 4-14%.[3–4]

Enuresis has been noted to be an under-reported problem and even parents of children with enuresis do not see it as a source of concern.[5] Epidemiology studies of enuresis across the world report varied prevalence rates amongst school children.[6–15] To the best of my knowledge there has been no report on enuresis amongst school children in Mumbai. It is seen by the author in his routine clinical practice that the problem of enuresis is prevalent in many primary school children.

The aim of the study was to examine the prevalence of enuresis in school children and to determine the contributing factors along with the treatment methods used in these children.

## MATERIALS AND METHODS

One thousand four hundred and seventy-three school children between the ages of 6-10 years studying in primary schools were the subjects of the study. Six different high schools were involved in the study.

A questionnaire for collecting the socio-demographic data, data with reference to enuresis and factors affecting it was prepared. The questionnaire had two parts – a part for enuretic children and a part for non-enuretic children. It was a small questionnaire that could be completed in a time span of around 10-15 min.

The purpose of the study was explained to the teachers and the questionnaire, a letter explaining the aims of the study and a consent form was sent in an enclosed envelope to the parents of the children. The study was approved by the Get Well Clinic ethics committee. Since this was an exploratory study and a cross-sectional survey, no form of intervention was planned for the children with enuresis. The questionnaire was designed to detect enuresis with predominantly psychological factors. The questionnaires were to be completed at home, the consent form to be duly signed and then returned to the teachers. The lost questionnaires were called for once to elicit a maximal response rate. Along with trying to attain a high response maximum attention was paid to avoid any embarrassment to the children. The questionnaire was based on a similar study performed in schools in Turkey.[16]

Enuresis was defined using the DSM-IV criteria of ‘bedwetting for at least two nights a week’.[17] Primary enuresis was defined as bedwetting in a child who had never had bladder control for a period longer than six months. Secondary enuresis was defined as enuresis seen in a child that was toilet trained for at least six months after the age of bladder control and with subsequent loss of bladder control. The DSM criteria make no provisions for the same. Questionnaires that were inconclusive and incomplete were excluded from the study. The entire data was analyzed by a qualified bio-statistician and the results were tabulated and presented.

## RESULTS

A total of 1698 questionnaires were administered out of which 1515 questionnaires were returned giving a response rate of 89.22%. Forty-two questionnaires were excluded as they were either incomplete or the data filled was inconclusive. A total of 1473 questionnaires were analyzed in the study. Of these 1086 questionnaires were answered by parents of male children while 387 were by parents of female children. This is in keeping with the fact that only two coeducation schools took part in the study and the other schools were boys' schools. The majority of the participants were between 7-10 years of age (86.15%) [Table 1].

**Table 1:** Age distribution of the children

Age group (years)	No. of children	Percentag
6	204	13.85
7	288	19.55
8	338	22.95
9	344	23.35
10	299	20.29

The prevalence of enuresis among boys was 9.02% while it was 3.61% among girls. The overall prevalence was 7.61% [Table 2].

**Table 2:** Prevalence of enuresis

Data	Males		Females		Total	
			
	N	%	N	%	N	%
Enuretics	98	87.5	14	12.5	112	100
Non enuretics	988	72.59	373	27.41	1361	100
Prevalence	9.02%	3.61%	7.61%			
Total	1086	78.7	387	21.3	1473	100

On exploring the characteristics of enuretic children it was noted that daytime incontinence was seen in 14.29%. This group was classified as voiding dysfunction and not enuresis and hence was excluded from the analysis in the study [Table 3]. The majority of the group had bedwetting every night (36.61%) while the rates of a positive family history for siblings and family members were 28.57% and 19.64% respectively [Table 4]. The majority of the parents expressed no concern over the problem of enuresis (62.5%) [Table 3]. Only 26.04% of the children were visiting a doctor.

**Table 3:** Parental concerns / treatment of enuresis

Data		Number of children N = 96	%
Parental concerns	No anxiety	60	62.5
	Worried	20	20.83
	High anxiety	16	16.66
Visiting a doctor	Yes	25	26.04
	No	71	73.96
Treatment (N = 25)	Drugs	19	76
	Combination	05	20
	Counselling	01	4
Result (N = 25)	No improvement	05	20
	Improvement	15	60
	Improvement and relapse	05	20
Using traditional methods* (N = 25)	Yes	20	80
	No	05	20

* Traditional methods means use of Imipramine, Desmopressin and bedwetting alarms

**Table 4:** Characteristics of the enuretic children

Data	Number of children (N = 112)		%
Frequency of bedwetting	Every night	41	36.61
	3–5 / week	23	20.54
	2 / week	24	21.43
	1 / week	16	14.29
	1 / month	08	7.14
Daytime bedwetting	Yes	16	14.29
	No	96	85.71
Dry night for > 6 months	Yes	09	8.04
	No	103	91.96
Did the children stop	Yes	46	41.07
Bedwetting if aroused	No	66	58.93
Family history In siblings	Yes	32	28.57
	No	80	71.43
Family history in	Yes	22	19.64
Parents / uncles	No	90	80.36

On comparing the characteristics of enuretic and non-enuretic children [Table 5], it was seen that lesser number of children in the enuretic group attained bladder control by the age of three years. Poor arousal (difficulty in making the child get up and pass urine) was in seen many enuretic children along with birth asphyxia, history of cesarean section, low birth-weight and absence of breastfeeding compared to non-enuretic children. Urinary tract infections along with daytime urgency were more common in the enuretic group. More children from the enuretic group lived with step-parents or had parents with health problems compared to the non-enuretic children.

**Table 5:** Characteristics of children with and without enuresis

Data	Enuretics		Non enuretic	
		
	N	% age	N	% age
Bladder control < 3 yrs	53	47.32	1121	82.37
Getting up by self	44	39.29	1051	77.21
Poor school performance	79	70.54	333	24.46
Daytime urgency	41	36.61	388	28.49
Urinary infection	31	27.68	106	7.78
Birth weight < 2500 g	09	8.04	68	5.86
Birth asphyxia	12	10.71	23	1.69
Cesarean section	51	45.54	522	46.56
Absence of breastfeeding	29	25.89	98	7.21
Large family	25	22.32	398	29.24
Low income	49	43.75	491	42.29
Living with step-parents / relatives	13	11.61	72	5.29
Parents with health problems	27	24.11	111	8.16
Low-education mothers	59	52.68	466	34.23
Low-education fathers	51	45.53	255	18.74

## DISCUSSION

Enuresis was more common in boys than girls. There are no precise definitions of enuresis. Various studies so far have used different criteria resulting in different prevalence being reported. A larger proportion of enuresis is usually the primary type. There are often no major differences in the factors contributing to enuresis whether primary or secondary.[18] The prevalence of enuresis reported in our study is in keeping with the range reported by three previous Indian studies on enuresis. In accordance with gender differences reported so far in various studies, here too we have a lower prevalence of enuresis amongst girls.

Arousal difficulty was reported more frequently in enuretic children supporting the fact that enuresis may result from dysfunctions in the nocturnal arousal response.[19]

From those visiting a doctor the majority received drug therapy and the majority was treated using the traditional methods that are used in the management of enuresis. An equal number of children amongst those receiving treatment had shown improvement, no improvement and improvement followed by a relapse.

Though drugs used in the management of enuresis were not studied it is common that the treatment of enuresis generally consisted of impiramine as that is the preferred drug amongst psychiatrists here. Behavioral interventions like bedwetting alarms are rarely used here in practice due to the easy use of medications and the fast response in many cases though alarm therapy has been proven to be successful in some studies.[20] Enuresis is a common problem among school children in Mumbai and needs attention. Very often parents do not report enuresis due to the embarrassment it may cause for the child and many parents assume that bladder control shall be attained over time. Teachers and parents in schools need to be educated to create an awareness and community insight into the problem.

In keeping with the proposed genetic basis of enuresis, many enuretic children did have a positive family history of enuresis.[21] Many children with enuresis had a history of daytime incontinence (inability to control urine) and urinary tract infection. No further information was obtained in this regard. A linear relationship between enuresis and the frequency of urinary tract infection has been noted in the past. There have been recent reports of enterobiasis infection in children with enuresis being the sole symptom at presentation.[22] Poor scholastic performance along with poor social adaptation is common in enuretic children. It may be both a result or a cause of enuresis.

Though enuresis is a single disorder and at times a single symptom, the causation is generally multifactorial and at times a complex interplay of physical and psychological factors is evident. When deciding treatment it often has to be multipronged keeping various factors in mind. The child as a whole along with his family and the environment he stays and studies in must be analyzed for the effective management of enuresis.
